# Mechanisms by which Bisphenol A affect the photosynthetic apparatus in cucumber (*Cucumis sativus* L.) leaves

**DOI:** 10.1038/s41598-018-22486-4

**Published:** 2018-03-09

**Authors:** Yu-Ting Li, Ying Liang, Yue-Nan Li, Xing-Kai Che, Shi-Jie Zhao, Zi-Shan Zhang, Hui-Yuan Gao

**Affiliations:** State Key Lab of Crop Biology, College of Life Sciences, Shandong Agricultural University, Tai’an, 271018 Shandong Province China

## Abstract

Bisphenol A (BPA), a widely distributed pollutant, suppresses photosynthesis in leaves. In previous studies on higher plants, the plants were treated by BPA through irrigation to root. This method cannot distinguish whether the BPA directly suppresses photosynthesis in leaves, or indirectly influences photosynthesis through affecting the function of root. Here, only the leaves but not the roots of cucumber were infiltrated with BPA solution. The photosystem II and I (PSII, PSI) were insensitive to BPA under darkness. BPA aggravated the PSII but not the PSI photoinhibition under light. BPA also inhibited CO_2_ assimilation, and the effect of BPA on PSII photoinhibition disappeared when the CO_2_ assimilation was blocked. The H_2_O_2_ accumulated in BPA-treated leaves under light. And the BPA-caused PSII photoinhibition was prevented under low (2%) O_2_. We also proved that the BPA-caused PSII photoinhibition depend on the turnover of D1 protein. In conclusion, this study proved that BPA could directly suppress photosynthesis in leaves, however, BPA does not damage PSII directly, but inhibits CO_2_ assimilation and over-reduces the electron transport chain under light, which increases the production of reactive oxygen species (H_2_O_2_), the over-accumulated ROS inhibits the turnover of D1 protein and consequently aggravates PSII photoinhibition.

## Introduction

Bisphenol A (2,2-bis (4-hydroxyphenyl) propane; BPA) is an intermediate compound used to produce epoxy resins, polycarbonates, polyesters, and coatings, and its global production is 5,500,000 tons per year in 2011^1^. Because of its mass production and wide use, BPA was largely discharged into the environment primarily through wastewater treatment plants, sewage sludge, and leachate from waste landfills^2,3^. BPA is ubiquitous in some natural environments because of continuous input, which has seriously threatened environmental safety and even endangered the balance of ecosystem.^4^

Plants are primary producers in ecosystem, they synthesize organic substances and provide energy for ecosystem. Some studies showed that the low concentrations of BPA promote the growth of soybean and carrot, however, high concentrations inhibited seed germination and root growth of broad bean, tomato, and durum wheat^5^. In addition, BPA obviously restrains the biosynthesis of chlorophyll^6^ and restrains photosynthesis^7^. It also decreases the biomass of shoot, leaf area and net photosynthetic rate^8^. Recent research reported that the BPA could cause DNA oxidative damage^9^.

In almost all the previous studies about the effects of BPA on plant, the plants were treated by BPA through irrigation to root^5,7,10–12^. BPA accumulated in roots, which seriously affected the growth and development of root^10–12^. Nie *et al*.^13^ observed that BPA solution irrigation inhibited respiratory metabolism related enzymes in root and affected the absorptive function of mineral elements in soybean seedling roots. The root supplies almost all water and mineral elements of leaves. The deficiency of provision of nutritious and water disturbs the growth and metabolism of shoot, especially the photosynthesis in leaves^14^. The damage of root by BPA may cause suppression of photosynthesis in leaves. In addition, the BPA absorbed by root can be transferred to shoot and it would enrich in the leaf blade, which maybe interfere with the photosynthesis in leaves. Therefore, the previous studies did not distinguish that BPA directly suppresses photosynthesis in leaves, or indirectly suppresses photosynthesis through damage of root.

In addition, former researches showed that there are several ways for the pollutants to enter leaf of plant^15,16^. The pollutants can be absorbed by root and transferred to leaf^17,18^. Furthermore, the pollutants can also settle from the atmosphere onto the leaf surface and then enter leaf through the waxy cuticle^15,16^. Due to losing the selective absorption of root and the blocking mechanism of root-stem transport, it is easier for the pollutant to enter leaf through the waxy cuticle accumulate in leaf^15,16^. Therefore, it is significant to verify if the BPA can interfere with photosynthesis in leaf directly.

The aim of the paper is to confirm that if the BPA could direct damage the photosynthetic apparatus in leaves independent of the root, and the mechanisms by which BPA affect the photosynthetic apparatus. It will expand our understanding of the impact of BPA on the plants, environmental and ecological. To explore whether the BPA has the direct effect on photosynthetic apparatus, the leaves were infiltrated with BPA solution. To verify the mechanisms by which BPA affects the photosynthesis, the leaves were infiltrated with BPA and exposed to high light under different environmental conditions to measure the activity of the photosynthetic, reactive oxygen species (ROS) content, and scavenging enzymes of ROS in leaves.

## Material and Methods

### Plant Materials

Cucumber (*Cucumis sativus* L. cultivar Jinchun 35) plants were grown in 0.35 L pots filled with rich soil. The plants were placed in a growth chamber at 25 °C/22 °C (day/night temperature) and 150 μmol m^−2^ s^−1^ of PPFD with a 14 h/10 h photoperiod and were supplied with sufficient water. We use the second leaves from the top of plants, youngest full developed leaves, on approximately 4-week-old plant in this experiments.

### Treatment

The leaves were infiltrated with 0.1, 0.2, 0.3 and 0.4 mmol concentration of BPA (contained 0.4% ethanol) or control solutions (0.4% ethanol solution) for 2 h under darkness. And then the leaves were placed in flowing air under darkness for 6 h to evaporate redundant water. Afterwards, the leaves were exposed to 800 μmol m^−2^ s^−1^ of PPFD light at 25 °C with 3 h for photoinhibition treatment.

To inhibit CO_2_ assimilation or D1 protein de novo synthesis, 1 mM iodacetamide (IAM)^19^ or 1 mM chloramphenicol (CM)^20,21^ were added during the infiltration.

A CIRAS-3 portable photosynthesis system (PP Systems, USA) was used to control the CO_2_ and O_2_ concentration of atmosphere during high light treatment. The 21% and 2% standard O_2_ gas were purchased from local supplier, and they were supplied into CIRAS-3 portable photosynthesis system from the air inlet.

### Chlorophyll fluorescence, 820 nm reflection and gas exchange

Chlorophyll fluorescence transient and 820 nm reflection were performed with dark-adapted leaves at room temperature by an M-PEA (Hansatech, UK). To obtain the W_K_ curve, the original chlorophyll fluorescence transient was standardized between O and J steps^22,23^. The chlorophyll fluorescence transients were analyzed with the JIP-test, the formulas of parameters are listed below^22,24^:

1. Absorption flux per cross section of leaf, ABS/CSm = Fm;
2. Maximum quantum yield of PSII, φ_Po_ = TR/ABS = Fv/Fm = 1 − (Fo/Fm);
3. The efficiency of electron moves beyond Q_A_^−^, ψ_Eo_ = ET/TR = 1 − V_j_;
4. The quantum yield for reduction of end electron acceptors at the PSI acceptor side, δ_Ro_ = RE/ET = (1 − V_i_)/(1 − V_j_).

The amplitude of 820-nm reflection (ΔI/Io) during far-red illumination was used to reflect the relative content of active PSI reaction center^25^.

The modulated chlorophyll fluorescence was measured with a FMS-2 pulse-modulated fluorometer (Hansatech, Norfolk, UK) according to the protocol described by Zhang *et al*.^26,27^. The light-adapted leaves were continuously illuminated by actinic light at 800 μmol m^−2^ s^−1^ from the FMS-2 light source, steady-state fluorescence (Fs) was recorded after 2 min illumination, and a 0.8 s saturating light of 8000 μmol m^−2^ s^−1^ was imposed to obtain the maximum fluorescence in the light-adapted state (Fm’). The actinic light was then turn off, and the minimum fluorenscence in the light-adapted state (Fo’) was determined by 3 s illumination with far-red light. The corresponding parameters were then calculated^28^.

1. Quantum yield of PSII, ΦPSII = (Fm’ − Fs)/Fm’;
2. Non-photochemical quenching, NPQ = (Fm − Fm’)/Fm’;
3. Photochemical quenching, qP = (Fm’ − Fs)/(Fm’ − Fo’);
4. Excitation energy capture by PSII reaction centers, Fv’/Fm’ = (Fm’ − Fo’)/Fm’.

The net photosynthetic rate (Pn) in leaves was measured using a CIRAS-3 portable photosynthesis system (PP Systems, Amsbury, MA, USA).The CO_2_ concentration, relative humidity, photon flux density and leaf temperature for measurements were maintained at 400 PPM, 60%, 800 μmol cm^−2^ s^−1^ of PPFD and 25^o^C via an automatic control device of CIRAS-3 photosynthesis system.

### Material content and enzyme activity

Leaf chlorophyll was extracted with 80% acetone in the dark for 72 h at 25 °C. The extracts were analyzed using an UV-visible spectrophotometer UV-1601 (Shimadzu, Japan) according the method of Parra^29^. H_2_O_2_ was extracted and determined according to the method of Patterson^30^. H_2_O_2_ content was calculated from a standard curve prepared by using different concentrations of H_2_O_2_ solutions.

*In situ* hydrogen peroxide (H_2_O_2_) was detected by DAB staining as previously described^31^. H_2_O_2_ reacts with DAB to form a reddish-brown stain. Treated leaf disks were incubated in DAB solution, PH 5.5, at 1 mg/ml. After incubation in the dark at room temperature for 20 h. Samples were boiled in alcohol (96%) for 10 min. After cooling, the leaf discs were extracted at room temperature with fresh ethanol and photographed. The starch was detected by I_2_-KI solution according to the methods of Ruzin^32^. The starch reacts with I_2_-KI solution to form a blue stain.

Leaf segments (0.2 g) were ground to a fine powder with liquid nitrogen and then homogenized in 2 ml of 50 mM potassium phosphate buffer (pH 7.8) containing 1 mM EDTA-Na_2_, 0.3% Triton X-100, and 1% (w/v) polyvinylpyrrolidone, with the addition of 1 mM ascorbate in the case of ascorbate peroxidase (APX) assays. The homogenate was centrifuged at 13,000 g for 20 min at 4 °C, and the supernatant were used for the following enzyme assays.

Superoxide dismutase (SOD) activity was assayed by monitoring the inhibition of the photochemical reduction of nitro blue tetrazolium (NBT) according to the methods of Giannopolitis and Ries^33^. APX activity was determined according to the method of Mishre, N.P.^34^ by monitoring the decrease in absorbance at 290 nm as ascorbate was oxidized (extinction coefficient of 2.8 mM^−1^ cm^−1^).

## Results and Disscussion

### The suppression of photosynthesis by BPA is independent of root

**Figure 1:**
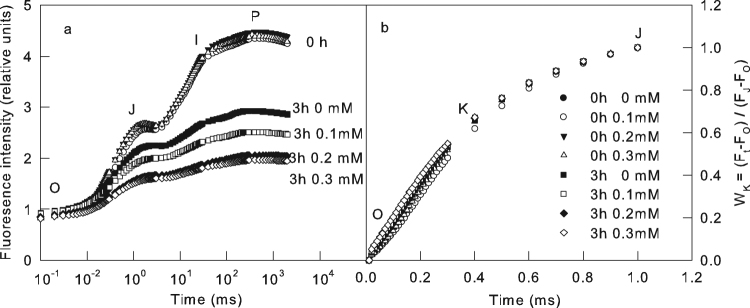
The effect of BPA on OJIP curve. The original chlorophyll a fluorescence transients (**a**), the W_K_ curves (**b**) obtained from the standardization between O and J steps in chlorophyll a fluorescence transients in cucumber leaves treated by different concentrations (0, 0.1, 0.2, 0.3 mM) of BPA after 0 h or 3 h high light treatment (800 μmol cm^−2^ s^−1^). Means ± SD, n = 6.

To eliminate the possible negative influence of BPA on root system and the indirect impress to the photosynthesis in leaves, we submerged the cucumber leaves into different concentrations of BPA solutions and water for 3 h in this study, and then the leaves were exposed to high light. Before high light treatment all of the chlorophyll fluorescence transient was measured to explore the change of photosynthetic apparatus, especially PSII activity. All of the chlorophyll a fluorescence transients showed a typical polyphasic rise with the basic steps of O-J-I-P (Fig. 1a). After high light treatment, the chlorophyll fluorescence transients had a significant change, and the change exacerbated with the increase of the BPA concentration (Fig. 1a, Supplementary Table S1), indicating the BPA aggravated the PSII photoinhibition under high light. This observation clarified that the BPA can impress the photosynthesis when applied on leaves, without the influence of root system.

### The damage of BPA to photosynthetic apparatus is selective

**Figure 2:**
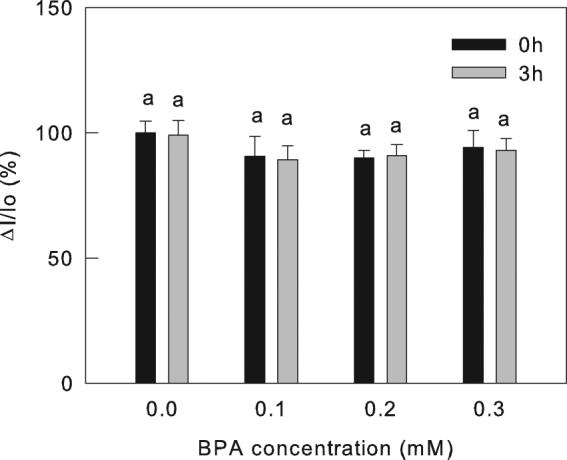
The effect of BPA on PSI activites. The amplitude of 820-nm reflection (ΔI/Io) during far-red illumination that reflectes the relative content of active PSI reaction center. The PSI activities were measured in cucumber leaves treated by different concentrations (0, 0.1, 0.2, 0.3 mM) of BPA after 0 h or 3 h high light treatment (800 μmol cm^−2^ s^−1^). Means ± SD, n = 6. Different letters indicate significant differences between different treatments (P < 0.05).

The PSI activities in control and BPA-treated leaves were analyzed by 820-nm transmission. The amplitude of 820-nm reflection (ΔI/Io) was used to reflect the relative content of active PSI RCs^25^. Although BPA obviously aggravated the PSII photoinhibition under high light, which was indicated by the change of the chlorophyll a fluorescence transients (Fig. 1a, Supplementary Table S1), however, the relative content of active PSI reaction centers was insensitive to BPA treatment (Fig. 2), which indicates that the damage of BPA to photosynthetic apparatus is selective.

In W_K_ curves obtained from the standardization between O and J steps in chlorophyll a fluorescence transients, the increase of K steps at 300 ms was considered as a specific indicator of injury to the donor side of PSII^22,23^. The BPA treatment did not obviously change the K steps in W_K_ curves before or after high light (Fig. 1b), which indicates that the BPA did not damage the donor side of PSII.

**Figure 3:**
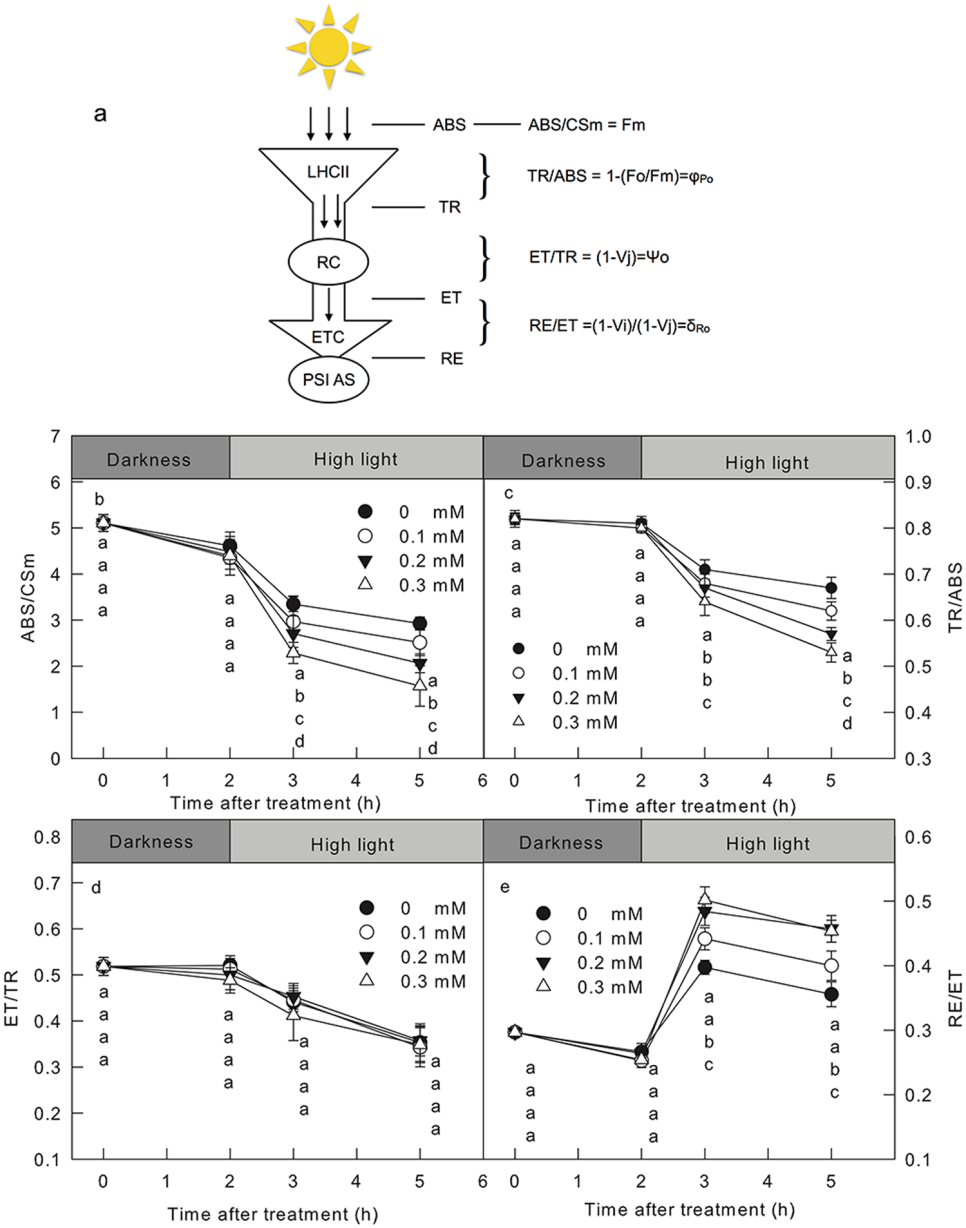
The effect of BPA on PSII activities. The diagrammatic sketch of JIP text (**a**); ABS: the photon flux absorbed by the pigments. PSI AS: PSI acceptor side. The absorption flux per cross section of leaf (ABS/CSm = Fm) (**b**); the maximum quantum yield of PSII (TR/ABS = φ_Po_ = Fv/Fm) (**c**); the efficiency of electron moves beyond Q_A_^−^ (ET/TR = ψ_Eo_ = 1 − V_j_) (**d**) and the quantum yield for reduction of end electron acceptors at the PSI acceptor side (RE/ET = δ_Ro_ = (1 − V_i_)/(1 − V_j_)) (**e**) in cucumber leaves treated by different concentrations (0, 0.1, 0.2, 0.3 mM) of BPA after 0 h or 3 h high light treatment (800 μmol cm^−2^ s^−1^). Means ± SD, n = 6. Different letters indicate significant differences between different treatments (P < 0.05).

To investigate further the inhibition site of BPA to PSII, the light absorption, energy transformation and electron transfer at the acceptor side of PSII were explored from the chlorophyll a fluorescence transient by JIP-test (Fig. 3)^22,24^. The absorption flux per cross section of leaf (ABS/CSm) gradually declined during the high light treatment, and it was lower in BPA-treated leaves than in control leaves (Fig. 3b). In addition, the decrease of ABS/CSm deteriorated with the increase of BPA concentration. The maximum quantum yield of PSII (Fv/Fm) that reflects the PSII photoinhibition and the efficiency of trapped energy in PSII^22,28^, changed similarly to the ABS/CSm (Fig. 3c). The efficiency of electron moves beyond Q_A_^−^ (ψ_Eo_), which reflects the capacity of electron transfer in the acceptor side of PSII^22,25^, decreased similarly after highlight treatment in all leaves treated by different concentrations of BPA (0–0.3 mM; Fig. 3d). In BPA treated leaves, the quantum yield for reduction of end electron acceptors at the PSI acceptor side (δ_Ro_), which reflects the electron transfer efficiency from Q_A_^−^ to NADP^+^ ^35,36^, and also usually use to reflect the activity of PSI complex^37,38^, increased with the raising of BPA concentration (Fig. 3e). Due to the ΔI/Io was maintained in BPA treated leaves, but the Fv/Fm decreased obviously, so the increase of δ_Ro_ was mainly caused by the decreased electron supply to ETC rather the improved PSI activity.

The above results proved that the BPA mainly inhibits the light absorption and light energy transformation of PSII, rather than the capacity of electron transfer in the acceptor or donor side of PSII or the activity of PSI.

**Figure 4:**
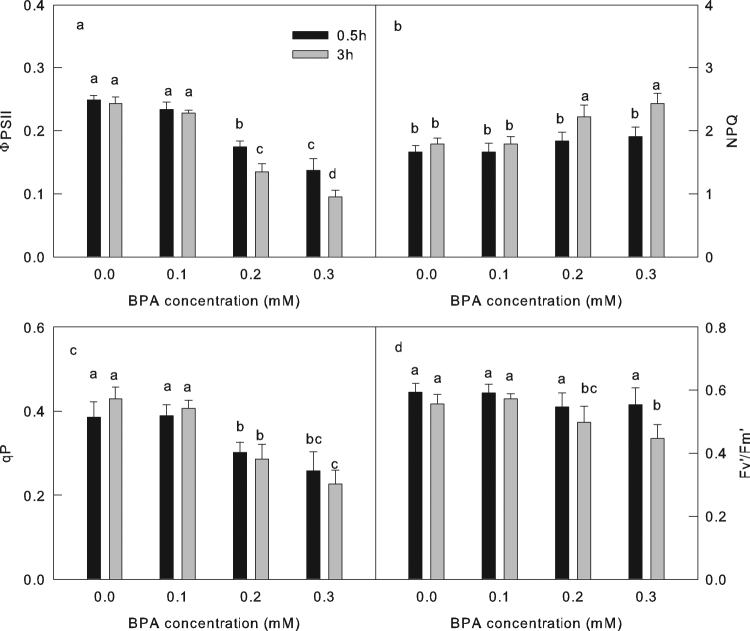
The effect of BPA on electron transfer of PSII. The quantum yield of electron transfers through PSII (ΦPSII) (**a**);non-photochemical quenching (NPQ)(**b**); photochemical quenching (qP) (**c**) and the efficiency of excitation energy captured by open PSII reaction centers (Fv’/Fm’)(**d**) in cucumber leaves treated by different concentrations (0, 0.1, 0.2, 0.3 mM) of BPA after 0.5 or 3 h high light treatment (800 μmol cm^−2^ s^−1^). Means ± SD, n = 6. Different letters indicate significant differences between different treatments (P < 0.05).

**Figure 5:**
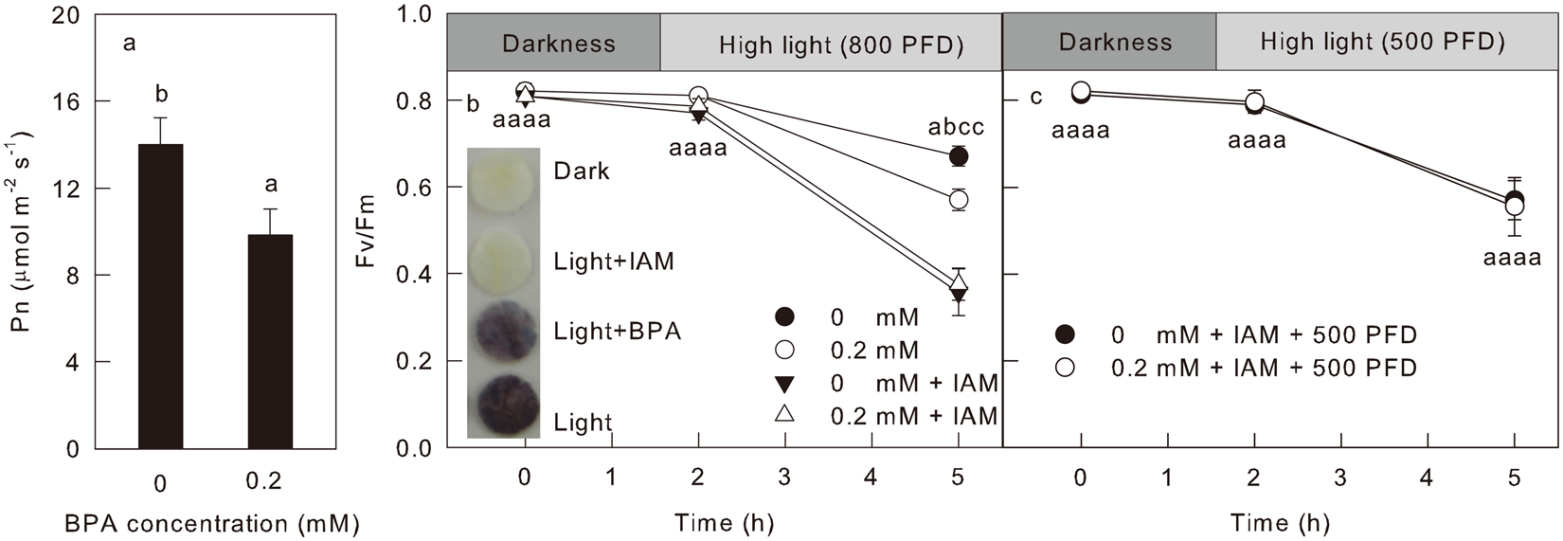
The effect of BPA on carbon assimilation. Plot (**a**) The net photosynthetic rate (Pn) in leaves treated by 0 or 0.2 mM BPA. Plot (**b**,**c**) the Fv/Fm in cucumber leaves treated by 0 or 0.2 mM BPA under darkness or high light (800 or 500 μmol m^−2 ^s^−1^) with or without presence of 1 mM IAM. Plot (**c**) insert, the histochemical detection of starch in leaves treated with 0.2 mM BPA or 1 mM iodoacetamide (IAM) under darkness or high light (800 μmol m^−2 ^s^−1^). Means ± SD, n = 6. Different letters indicate significant differences between different treatments (P < 0.05).

Previous research reported that the long-term leaf irrigation with BPA solution decreases chlorophyll content of leaves^6,7^. In this study, there is no significant change in chlorophyll content among the different treatments (Table 1), which indicates that the inactivation of photosynthetic apparatus (Figs 1–5) caused by BPA is independent of the degradation of pigments.

**Table 1:** Total chlorophyll (Chl a + b) content and the ratio between chlorophyll a and chlorophyll b (Chl a/b) in leaves treated with different concentration BPA (0, 0.1, 0.2 or 0.3 mM BPA) before (0 h) and 3 h after high light treatment (800 μmol m^−2^ s^−1^).

	Chl a + b	Chl a/b
0 h	14.64 ± 0.34 a	2.63 ± 0.16 a
0 mM	15.23 ± 0.29 a	2.53 ± 0.14 a
0.1 mM	15.24 ± 0.56 a	2.56 ± 0.24 a
0.2 mM	15.22 ± 0.41 a	2.48 ± 0.28 a
0.3 mM	15.46 ± 0.40 a	2.50 ± 0.21 a

Means ± SD, n = 6. Different letters indicate significant differences between different treatments (P < 0.05).

### The mechanism under PSII photoinhibition caused by BPA: the inhibition of CO_2_ fixation interferes electron transfer

The BPA aggravated PSII photoinhibition under high light but it did not affect the activity of PSII in darkness (Fig. 1), which indicates that BPA do not injure the photosynthetic apparatus directly but the BPA-caused photoinhibition depend on the light.

Under high light, the BPA treatment sharply decreased the quantum yield of electron transfer through PSII (ΦPSII) in cucumber leaves, even at the beginning of high light (30 min; Fig. 4a), so the low ΦPSII in BPA treated leaves is not the result, but the cause of PSII photoinhibition. In addition, the non-photochemical quenching (NPQ), which is one of the most important PSII photoprotection mechanisms^39^, was higher in BPA treated leaves than that in the control leaves (Fig. 4b). The results indicate that the activation of PSII photoprotection mechanism did not eliminate the negative effect of BPA on PSII.

Moreover, the photochemical quenching of PSII (qP) changed similarly to the ΦPSII under high light (Fig. 4c). In contrast, the efficiency of excitation energy captured by open PSII reaction centers (Fv’/Fm’) in control leaves was similar with that in leaves treated with low concentration of BPA (0.1 and 0.2 mM), and slightly higher than that in the leaves treated with 0.3 mM BPA after 3 h high light treatment (Fig. 4d). The ΦPSII calculated as Fv’/Fm’ multiplied by qP, the later indicates the opening degree of electron transport chain (ETC)^28^. The observation is that the decrease of ΦPSII in BPA treated leaves was mainly due to the decline of the qP (Fig. 4), which reflects that the inhibition of electron transfer lead to the decrease of ΦPSII. Previous discussion has showed that the BPA did not impress the capacity of electron transfer in the acceptor or donor side of PSII (Fig. 1b), so the block of ETC in BPA treated leaves under high light is not due to the damage of electron transfer itself. Almost all of the products of the photosynthetic electron transfer, ATP and NADPH, were consumed by CO_2_ fixation. The inhibition of CO_2_ fixation increased the accumulation of ATP and NADPH, which caused a feedback inhibition of photosynthetic electron transport. Therefore, we speculate that the block of ETC was attributed by the inactive CO_2_ fixation that locates downstream of ETC in BPA treated leaves.

Consistent with our speculation, the 0.2 mM BPA remarkably decreased the net photosynthetic CO_2_ assimilation rate (Pn) (Fig. 5a). To explore the contribution of the inhibition of CO_2_ assimilation to the BPA-caused PSII photoinhibition, we pre-treated the leaves by IAM, an inhibitor of the Calvin cycle^19^, together with 0.2 mM BPA, and then exposed the leaves to high light. The presence of IAM greatly increased PSII photoinhibition. To avoid that the effect of BPA on PSII photoinhibiton was covered up by the severe PSII photoinhibiton with the presence of IAM, a weaker light (500 μmol m^−2^.s^−1^) was used instead of 800 μmol m^−2^.s^−1^ light to illuminate leaves. The IAM prevented the accumulation of starch in leaves (Fig. 5b insert), which indicates the CO_2_ fixation was completely blocked. Moreover, IAM abolished the decrease of Fv/Fm caused by BPA, which is independent of light intensity (Fig. 5b,c). These results support the above speculation that the BPA interferes electron transfer and aggravates PSII photoinhibition through inactive CO_2_ fixation.

### The mechanism under PSII photoinhibition caused by BPA: ROS accumulation inhibits the repair of photodamaged PSII

**Figure 6:**
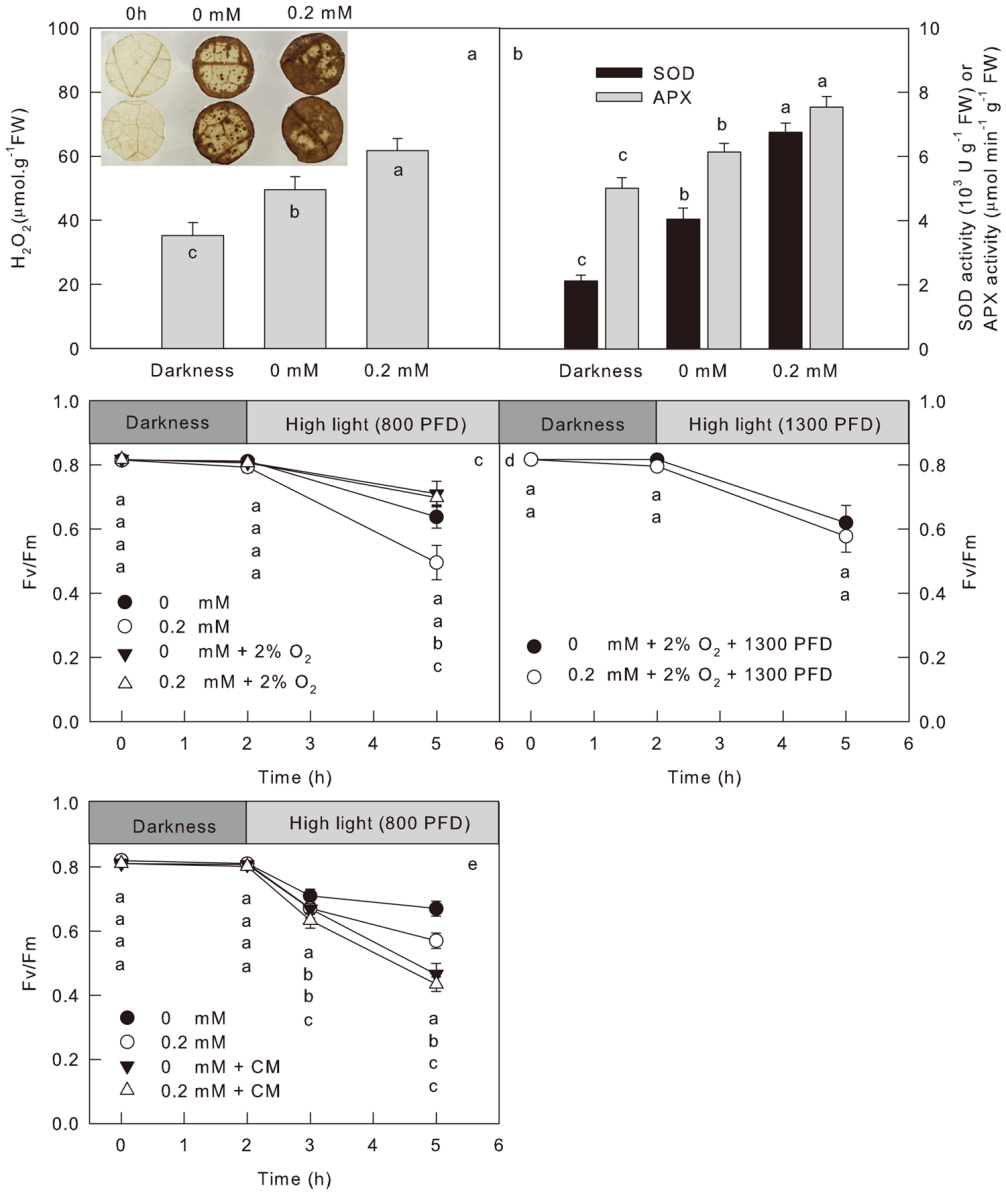
The effect of BPA on ROS. Plot (**a**–**d)** the quantitative determination (**a**) or histochemical detection (insert in plot a) of hydrogen peroxide (H_2_O_2_), as well as the activities of SOD and APX (**b**) in cucumber leaves treated by 0 or 0.2 mM BPA before (0 h) or after 3 h high light treatment (800 μmol cm^−2^ s^−1^). Plot (**c**,**d**) the Fv/Fm in cucumber leaves treated by 0 or 0.2 mM BPA under darkness or high light (800 or 1300 μmol m^−2 ^s^−1^) under 21% or 2% O_2_. Plot (**e**) the Fv/Fm in cucumber leaves treated by 0 or 0.2 mM BPA under darkness or high light treatment (800 μmol cm^−2^ s^−1^) with or without the presence of chloramphenicol (CM), an inhibitor of D1 protein synthesis. Means ± SD, n = 6. Different letters indicate significant differences between different treatments (P < 0.05).

Photosynthetic electron transport chain is the main source of reactive oxygen species (ROS) in leaves under light, and it is the primary target to attack ROS^40^. There are two major sites of ROS generation in ETC: the end of ETC (acceptor side of PSI) and the PSII reaction centers^40^. Many studies have verified that the interference of electron transfer will result in the over production of ROS^41,42^. To study the influence of BPA on ROS in leaves, the accumulation of hydrogen peroxide (H_2_O_2_), the most stable ROS, was detected by quantitative determination or histochemical detection (Fig. 6a and insert). Both the two experiments showed that the contents of H_2_O_2_ in leaves increased significantly after high light treatment, but the increase was more remarkable in BPA treated leaves than in control leaves. Moreover, the activities of SOD and APX, the two of the most important ROS scavenging enzymes, significantly increased in BPA treated leaves (Fig. 6b), which indicates that the over-accumulation of ROS in BPA-treated leaves was not due to the inactivation of ROS scavenging system but due to the stimulation of ROS generation by BPA.

The low O_2_ atmosphere should restrain the generation of ROS. To analyze further the contribution of ROS accumulation to the PSII photoinhibition caused by BPA, the leaves were illuminated by high light under low O_2_ (2%). Because the PSII photoinhibition was much lower under 2% O_2_ than that under 21% O_2_, a stronger light (1300 μmol.m^−2^.s^−1^) was used to illuminate leaves under low O_2_. Regardless of light intensity, the difference between the Fv/Fm in BPA-treated leaves and not treated leaves was disappeared under low O_2_ (Fig. 6c,d). These factors proved that the over-accumulation of ROS due to the repressed CO_2_ fixation and electron transfer in BPA treated leaves is essential in the BPA-caused PSII photoinhibition.

ROS can cause PSII photoinhibition by two ways: damaging PSII directly^43–46^, or inhibiting the repair of photo-damaged PSII at the step of de-novo synthesis of the D1 protein and therefore aggravates PSII photoinhibition^47–50^. To distinguish the effect of BPA on the photodamage of PSII and the inhibition of photo-damaged PSII repair, cucumber leaves were pretreated with CM before the high light treatment. The CM is an inhibitor of D1 protein synthesis, it can inhibit the repair of the photodamaged PSII^20,21^. It was observed that the BPA treatment did not accelerate the decrease of Fv/Fm in the presence of CM, but it did accelerate photoinhibition in the absence of CM (Fig. 6e). This observation indicates that the accumulation of ROS aggravates PSII photoinhibition in BPA treated leaves mainly via inhibition of the photodamaged PSII repair rather than by damaging PSII directly.

## Conclusion

The present research proved that BPA significantly damages the photosynthetic apparatus without the contribution of roots. BPA does not directly damages the photosynthetic electron transport chain. It mainly inhibits the carbon assimilation, therefore it leads to the over-production of the electron transfer chain and over-excitation of PSII reaction centers to induce the over-production of ROS under high light conditions. The over-accumulation of ROS inhibits the repair of photo-damaged PSII and then it aggravates the PSII photoinhibition under high light.

## Electronic supplementary material

Supplementary Table S1
