# Supplementary material for: Mechanisms by which Bisphenol A affect the photosynthetic apparatus in cucumber (Cucumis sativus L.) leaves
Source: Sci Rep. 2018 Mar 9;8:4253. doi: 10.1038/s41598-018-22486-4 (PMC5844896; doi:10.1038/s41598-018-22486-4)
Supplement: Supplementary file 1 — Supplementary Table S1 [file 41598_2018_22486_MOESM1_ESM.pdf]

Title: Mechanisms by which Bisphenol A affect the photosynthetic apparatus in cucumber (*Cucumis sativus* L.) leaves

Yu-Ting Li, Ying Liang , Yue-Nan Li, Xing-Kai Che, Shi-Jie Zhao, Zi-Shan Zhang*, Hui-Yuan Gao

State Key Lab of Crop Biology, College of Life Sciences, Shandong Agricultural University, Tai'an, 271018, Shandong Province, China

*Corresponding author: Zi-shan Zhang; Telephone number: 86 538 8245985; E-mail address: zhangzishantaian@163.com

Supplementary Table S1: The fluorescence intensity in O, K, J, I, P steps of chlorophyll fluorescence transients (Fo, Fk, Fj, Fi, Fp) in leaves treated with different concentration BPA (0, 0.1, 0.2 or 0.3mM BPA) before and 3h after high light treatment (800 μmol m^{-2} s^{-1}). Means ± SD, n=6.

Concentration （mM）	Fo	Fk	Fj	Fi	Fp
0—0	8528.6±505.80	16500±1194.66	26734±1336.53	38581±1886.02	46122.6±2982.33
0—0.1	8419.6±368.30	16052±822.55	25715.2±1603.86	36462.2±2231.10	43510.6±2480.07
0—0.2	8318.6±504.86	17406.6±1065.76	26719.8±861.34	37170.2±1722.00	44845.8±2652.44
0—0.3	8254±377.62	17190.2±1187.14	26650±2062.89	36961±3725.43	43941±4207.31
3—0	9249.1±385.71	15505.3±882.21	22372.6±1246.91	25810.9±1334.22	29257.9±1408.01
3—0.1	9126.4±578.46	14329.9±1370.57	19824.7±2344.23	22446.4±2720.52	25112.1±2840.43
3—0.2	8529.5±479.74	12370.7±1050.85	16468.4±1615.00	18184.6±1874.03	20615.2±2037.65
3—0.3	8221.9±883.36	11969.5±2197.94	15893.6±3441.40	17549.5±4126.22	19689.8±4404.92
